# Draft genome sequence of *Streptomyces* sp. MWW064 for elucidating the rakicidin biosynthetic pathway

**DOI:** 10.1186/s40793-016-0205-3

**Published:** 2016-10-21

**Authors:** Hisayuki Komaki, Arisa Ishikawa, Natsuko Ichikawa, Akira Hosoyama, Moriyuki Hamada, Enjuro Harunari, Takuya Nihira, Watanalai Panbangred, Yasuhiro Igarashi

**Affiliations:** 1 Biological Resource Center, National Institute of Technology and Evaluation (NBRC), Chiba, Japan; 2 Biotechnology Research Center and Department of Biotechnology, Toyama Prefectural University, Toyama, Japan; 3 NBRC, Tokyo, Japan; 4 International Center for Biotechnology, Osaka University, Osaka, Japan; 5 Mahidol University and Osaka University Collaborative Research Center on Bioscience and Biotechnology, Bangkok, Thailand; 6 Department of Biotechnology, Faculty of Science, Mahidol University, Bangkok, Thailand

**Keywords:** Biosynthesis, Nonribosomal peptide synthetase, Polyketide synthase, Rakicidin, *Streptomyces*

## Abstract

*Streptomyces* sp. MWW064 (=NBRC 110611) produces an antitumor cyclic depsipeptide rakicidin D. Here, we report the draft genome sequence of this strain together with features of the organism and generation, annotation and analysis of the genome sequence. The 7.9 Mb genome of *Streptomyces* sp. MWW064 encoded 7,135 putative ORFs, of which 6,044 were assigned with COG categories. The genome harbored at least three type I polyketide synthase (PKS) gene clusters, seven nonribosomal peptide synthetase (NRPS) gene clusters, and four hybrid PKS/NRPS gene clusters, from which a hybrid PKS/NRPS gene cluster responsible for rakicidin synthesis was successfully identified. We propose the biosynthetic pathway based on bioinformatic analysis, and experimentally proved that the pentadienoyl unit in rakicidins is derived from serine and malonate.

## Introduction

Rakicidin D is an inhibitor of tumor cell invasion isolated from the culture broth of an actinomycete strain MWW064 of the genus *Streptomyces* [1]. To date, five congeners rakicidins A, B, and E from *Micromonospora* and rakicidins C and D from *Streptomyces* have been reported [1–4]. Rakicidins share the 15-membered cyclic depsipeptide structure comprising three amino acids and a fatty acid modified with hydroxy and methyl substitutions. The most intriguing part of rakicidins is a rare unusual amino acid, 4-amino-2,4-pentadienoate (APDA), which is present only in a limited range of secondary metabolites of actinomycetes such as BE-43547 [5] and microtermolide [6, 7]. Despite the scarcity of APDA unit in nature, nothing is known about its biosynthesis. Recently, putative biosynthetic genes for rakicidin D were reported [8], but the data is incomplete, no detailed information is shown in the paper, and DNA sequences have not been registered in public databases. Hence, the biosynthesis of rakicidins has been actually unclear yet. In this study, we performed whole genome shotgun sequencing of the strain MWW064 to elucidate the biosynthetic mechanism of rakicidin D. We herein present the draft genome sequence of *Streptomyces* sp. MWW064, together with the taxonomical identification of the strain, description of its genome properties and annotation of the gene cluster for rakicidin synthesis. We propose the rakicidin-biosynthetic mechanism predicted by bioinformatics analysis and confirmed by precursor-incorporation experiments.

## Organism information

### Classification and features

**Fig. 1:**
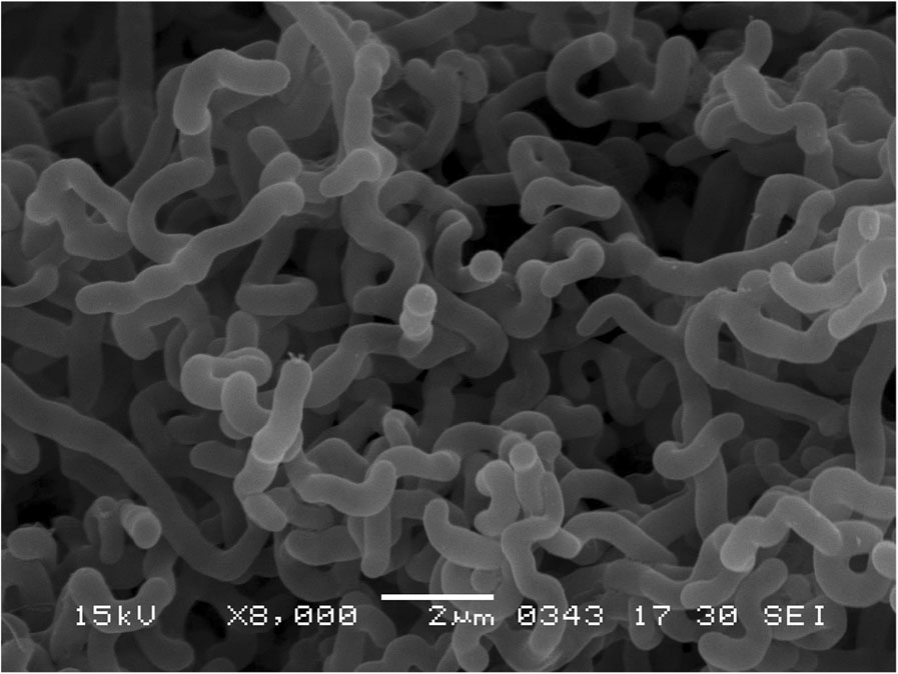
Scanning electron micrograph of *Streptomyces* sp. MWW064 grown on 1/2 ISP 2 agar for 7 days at 28 °C. Bar, 2 μm

**Fig. 2:**
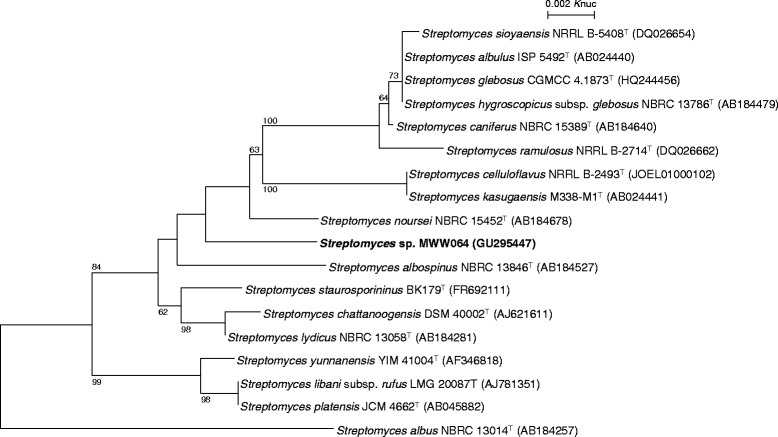
Phylogenetic tree of *Streptomyces* sp. MWW064 and phylogenetically close type strains, showing over 98.5 % similarity, based on 16S rRNA gene sequences. The accession numbers for 16S rRNA genes are shown in parentheses. The tree uses sequences aligned by ClustalX2 [9], and constructed by the neighbor-joining method [35]. All positions containing gaps were eliminated. The building of the tree also involves a bootstrapping process repeated 1,000 times to generate a majority consensus tree, and only bootstrap values above 50 % are shown at branching points. *Streptomyces albus* NBRC 13014^T^ was used as an outgroup

In the course of screening for antitumor compounds from actinomycetes, *Streptomyces* sp. MWW064 was isolated from a marine sediment sample collected in Samut Sakhon province of Thailand and found to produce rakicidin D [1]. The general feature of this strain is shown in Table 1. This strain grew well on ISP 2 and ISP 4 agars. On ISP 5 and ISP 7 agars, the growth was poor. The color of aerial mycelia was white and that of the reverse side was pale red on ISP 2 agar. Diffusible pigments were dark orange on ISP 2 agar medium. Strain MWW064 formed extensively branched- substrate and aerial mycelia. The aerial mycelium formed flexuous spore chains at maturity. The spores were cylindrical, having a smooth surface. A scanning electron micrograph of this strain is shown in Fig. 1. Growth occurred at 15–37 °C (optimum 28 °C) and pH 5–9 (optimum pH 7). Strain MWW064 exhibited growth with 0–3 % (w/v) NaCl (optimum 0 % NaCl). Strain MWW064 utilized glucose and inositol for growth. The gene sequence encoding 16S rRNA was obtained from GenBank/EMBL/DDBJ databases (accession no. GU295447). A phylogenetic tree was reconstructed on the basis of the 16S rRNA gene sequence together with taxonomically close *Streptomyces* type strains using ClustalX2 [9] and NJPlot [10]. The phylogenetic analysis confirmed that the strain MWW064 belongs to the genus *Streptomyces* (Fig. 2).

**Table 1:** Classification and general features of *Streptomyces* sp. MWW064 [13]

MIGS ID	Property	Term	Evidence code^a^
	Classification	Domain *Bacteria*	TAS [24]
		Phylum *Actinobacteria*	TAS [25]
		Class *Actinobacteria*	TAS [26]
		Order *Actinomycetales*	TAS [26–29]
		Suborder *Streptomycineae*	TAS [26, 29]
		Family *Streptomycetaceae*	TAS [26, 28–31]
		Genus *Streptomyces*	TAS [28, 31–33]
		Species undetermined	-
		strain: MWW064	TAS [1]
	Gram stain	Gram-positive	NAS
	Cell shape	Branched mycelia	IDA
	Motility	Not reported	
	Sporulation	Sporulating	IDA
	Temperature range	15 °C to 37 °C	IDA
	Optimum temperature	28 °C	IDA
	pH range; Optimum	5 to 9; 7	IDA
	Carbon source	D-glucose, inositol	IDA
MIGS-6	Habitat	Marine sediment	TAS [1]
MIGS-6.3	Salinity	0 % to 3 % NaCl	IDA
MIGS-22	Oxygen requirement	Aerobic	IDA
MIGS-15	Biotic relationship	Free-living	IDA
MIGS-14	Pathogenicity	Not reported	
MIGS-4	Geographic location	Samut Sakhon province, Thailand	TAS [1]
MIGS-5	Sample collection	February 2, 2008	NAS
MIGS-4.1	Latitude	13° 32’ 55” N	NAS
MIGS-4.2	Longitude	100° 16’ 39” E	NAS
MIGS-4.4	Altitude	8.6 m. above sea level	NAS

^a^ Evidence codes - IDA: Inferred from Direct Assay; TAS: Traceable Author Statement (i.e., a direct report exists in the literature); NAS: Non-traceable Author Statement (i.e., not directly observed for the living, isolated sample, but based on a generally accepted property for the species, or anecdotal evidence). These evidence codes are from the Gene Ontology project [34]

#### Chemotaxonomic data

The isomer of diaminopimelic acid in the whole-cell hydrolysate was analyzed according to the method described by Hasegawa et al. [11]. Isoprenoid quinones and cellular fatty acids were analyzed as described previously [12]. The whole-cell hydrolysate of strain MWW064 contained ll-diaminopimelic acid as its diagnostic peptidoglycan diamino acid. The predominant menaquinones were identified as MK-9(H_2_), MK-9(H_4_) and MK-9(H_6_); MK-10(H_2_), MK-10(H_4_) and MK-10(H_6_) were also detected as minor components. The major cellular fatty acids were found to be anteiso-C_15:0_, iso-C_15:0_, C_16:0_, anteiso-C_17:0_, iso-C_17:0_ and iso-C_16:0_.

## Genome sequencing information

### Genome project history

In collaboration between Toyama Prefectural University and NBRC, the organism was selected for genome sequencing to elucidate the rakicidin biosynthetic pathway. We successfully accomplished the genome project of *Streptomyces* sp. MWW064 as reported in this paper. The draft genome sequences have been deposited in the INSDC database under the accession number BBUY01000001-BBUY01000099. The project information and its association with MIGS version 2.0 compliance are summarized in Table 2 [13].

**Table 2:** Project information

MIGS ID	Property	Term
MIGS 31	Finishing quality	Improved-high-quality draft
MIGS-28	Libraries used	454 shotgun library, Illumina paired-end library
MIGS 29	Sequencing platforms	454 GS FLX+, Illumina HiSeq1000
MIGS 31.2	Fold coverage	8.9 ×, 93.5 ×, respectively
MIGS 30	Assemblers	Newbler v2.8, GenoFinisher
MIGS 32	Gene calling method	Progidal
	Locus Tag	SSP35
	GenBank ID	BBUY00000000
	GenBank Date of Release	February 20, 2016
	GOLD ID	Not registered
	BIOPROJECT	PRJDB3538
MIGS 13	Source Material Identifier	NBRC 110611
	Project relevance	Industrial

### Growth conditions and genomic DNA preparation

*Streptomyces* sp. MWW064 was deposited in the NBRC culture collection with the registration number of NBRC 110611. Its monoisolate was grown on polycarbonate membrane filter (Advantec) on double diluted ISP 2 agar medium (0.2 % yeast extract, 0.5 % malt extract, 0.2 % glucose, 2 % agar, pH 7.3) at 28 °C. High quality genomic DNA for sequencing was isolated from the mycelia with an EZ1 DNA Tissue Kit and a Bio Robot EZ1 (Qiagen) according to the protocol for extraction of nucleic acid from Gram-positive bacteria. The size, purity, and double-strand DNA concentration of the genomic DNA were measured by pulsed-field gel electrophoresis, ratio of absorbance values at 260 nm and 280 nm, and Quant-iT PicoGreen dsDNA Assay Kit (Life Technologies), respectively, to assess the quality of genomic DNA.

### Genome sequencing and assembly

Shotgun and paired-end libraries were prepared and subsequently sequenced using 454 pyrosequencing technology and HiSeq1000 (Illumina) paired-end technology, respectively (Table 2). The 70 Mb shotgun sequences and 739 Mb paired-end sequences were assembled using Newbler v2.8 and subsequently finished using GenoFinisher [14] to yield 99 scaffolds larger than 500 bp.

### Genome annotation

Coding sequences were predicted by Prodigal [15] and tRNA-scanSE [16]. The gene functions were annotated using an in-house genome annotation pipeline, and PKS- and NRPS-related domains were searched using the SMART and PFAM domain databases. PKS and NRPS gene clusters and their domain organizations were determined as reported previously [17]. Substrates of adenylation (A) and acyltransferase (AT) domains were predicted using antiSMASH [18]. BLASTP search against the NCBI nr databases were also used for predicting function of proteins encoded in the rakicidin biosynthetic gene cluster.

## Genome properties

The total size of the genome is 7,870,697 bp and the GC content is 71.1 % (Table 3), similar to other genome-sequenced *Streptomyces* members. Of the total 7,206 genes, 7,135 are protein-coding genes and 71 are RNA genes. The classification of genes into COGs functional categories is shown in Table 4. As for secondary metabolite pathways by modular PKSs and NRPSs, *Streptomyces* sp. MWW064 has at least four hybrid PKS/NRPS gene clusters, three type I PKS gene clusters, and seven NRPS gene clusters. According to the assembly line mechanism [19], we predicted the chemical backbones that each cluster will synthesize (Table 5), suggesting the potential of *Streptomyces* sp. MWW064 to produce diverse polyketide- and nonribosomal peptide-compounds as the secondary metabolites.

**Table 3:** Genome statistics

Attribute	Value	% of Total
Genome size (bp)	7,904,619	100.0
DNA coding (bp)	6,855,885	86.7
DNA G + C (bp)	5,597,799	70.8
DNA scaffolds	99	-
Total genes	7,206	-
Protein coding genes	7,135	99.0
RNA genes	71	0.99
Pseudogenes	-	-
Genes in internal clusters	2,610	36.2
Genes with function prediction	4,515	62.7
Genes assigned to COGs	6,044	83.9
Genes with Pfam domains	4,870	67.6
Genes with signal peptides	559	7.8
Genes with transmembrane helices	1,550	21.5
CRISPR repeats	1	-

**Table 4:** Number of genes associated with general COG functional categories

Code	Value	% age	Description
J	279	4.6	Translation, ribosomal structure and biogenesis
A	4	0.1	RNA processing and modification
K	696	11.5	Transcription
L	452	7.5	Replication, recombination and repair
B	6	0.1	Chromatin structure and dynamics
D	55	0.9	Cell cycle control, Cell division, chromosome partitioning
V	132	2.2	Defense mechanisms
T	432	7.1	Signal transduction mechanisms
M	294	4.9	Cell wall/membrane biogenesis
N	33	0.5	Cell motility
U	95	1.6	Intracellular trafficking and secretion
O	223	3.7	Posttranslational modification, protein turnover, chaperones
C	386	6.4	Energy production and conversion
G	474	7.8	Carbohydrate transport and metabolism
E	651	10.8	Amino acid transport and metabolism
F	134	2.2	Nucleotide transport and metabolism
H	253	4.2	Coenzyme transport and metabolism
I	323	5.3	Lipid transport and metabolism
P	404	6.7	Inorganic ion transport and metabolism
Q	385	6.4	Secondary metabolites biosynthesis, transport and catabolism
R	1,032	17.1	General function prediction only
S	440	7.3	Function unknown
-	1,091	18.1	Not in COGs

The total is based on the total number of protein coding genes in the genome

**Table 5:** Modular PKS and NRPS gene clusters in *Streptomyces* sp. MWW064

Gene cluster	Encoded in	No. of modular PKS and NRPS genes	No. of modules	Backbone of predicted product
*pks/nrps-1 (rak)*	scaffold 9	6	7	R-C_3_-C_3_-Ser-C_2_-Gly-X
*pks/nrps-2*	scaffold 5	6	14	C_2_-C_2_-C_2_-C_2_-C_2_-Gly-C_2_-C_2_-C_2_-C_2_-C_2_-C_2_-C_2_-C_2_
*pks/nrps-3*	scaffold 2	4	3	C_?_-C_?_-X
*pks/nrps-4*	scaffold 11	1	2	X-C_2_
*pks-1*	scaffold 18	5	5	C_?_-C_3_-C_2_-C_2_-C_?_
*pks-2*	scaffold 23	1	1	C_?_
other *pks* ^a^	scaffolds 11, 39, 45	>3	>10	C_2_-C_2_-C_3_-C_2_, C_2_-C_2_, C_2_-C_2_-C_2_, C_2_
*nrps-1*	scaffold 11	4	4	X-X-Val-X
*nrps-2*	scaffold 18	3	3	R-Val-X
*nrps-3*	scaffold 9	2	3	R-Cys-mCys
*nrps-4*	scaffold 13	3	4	Val-Gly-Ser-Pro
*nrps-5*	scaffold 2	1	1	Ser
*nrps-6*	scaffold 12	1	1	Thr
other *nrps* ^a^	scaffolds 3, 5	>2	>6	X-X-X-X-X, Cys

^a^not completely sequenced. R, starter unit; C_3_, C_3_ unit derived from methylmalonyl-CoA; C_2_, C_2_ unit derived from malonyl-CoA; X, unpredictable amino acid; C_?_, unpredictable carbon unit derived from acyl-CoA; mCys, methylated cysteine

## Insights from the genome sequence

### Rakicidin biosynthetic pathway in *Streptomyces* sp. MWW064

**Fig. 3:**
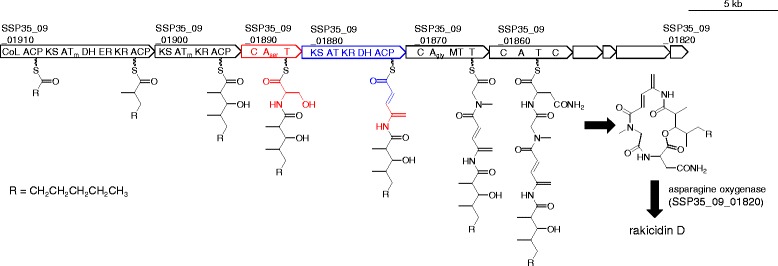
Genetic map of rakicidin biosynthetic gene cluster of *Streptomyces* sp. MWW064 and the biosynthetic mechanism of rakicidin D

The chemical structure of rakicidin D suggested that it is synthesized by a hybrid PKS/NRPS pathway. Among the four hybrid PKS/NRPS gene clusters present in *Streptomyces* sp. MWW064 (Table 5), *pks/nrps-1* is most likely responsible for rakicidin synthesis because the carbon backbone of the predicted product (R-C_3_-C_3_-Ser-C_2_-Gly-X) is in good accordance with that of rakicidin D. Genes in *pks/nrps-1* (Table 6) encode enzymes necessary for rakicidin biosynthesis (Fig. 3). This cluster contains three PKS genes (SSP35_09_01910, SSP35_09_01900, SSP35_09_01880) and three NRPS genes (SSP35_09_01890, SSP35_09_01870, SSP35_09_01860), corresponding to *rakAB*, *rakC*, *rakEF*, *rakD*, *rakG*, and *rakH* [8], respectively. Based on the collinearity rule of modular PKS/NRPS pathways, it is deduced that RakAB loads a starter molecule (‘R’ in Fig. 3), and subsequently RakAB and RakC add a diketide chain to the starter by condensation of two methylmalonyl-CoA molecules, since the substrates of their AT domains are likely methylmalonyl-CoA (‘AT_m_’ in Fig. 3). An NRPS RakD and the remaining PKS RakEF are most likely involved in the APDA supply: the A domain of RakD has signature amino acid residues for serine, and RakEF contains a set of domains (AT, KR, DH) for malonate incorporation, ketoreduction, and dehydration to provide a double bond between C9 and C10. In addition, the DH domain in RakEF is also proposed to be responsible for the dehydration of the primary hydroxy group of the incorporated serine molecule on the basis of the following reasons although experimental evidences are required. First, no dehydratase gene is present near the rakicidin cluster. In the biosynthesis of dehydroalanine in bacterial peptides such as lantibiotics, a dehydratase catalyzes the *exo*-methylene formation from serine [20, 21]. Second, the order of KR and DH domains in RakEF is unusual: among the three hundred type I PKS genes for eighty actinomycete polyketides, the order of two domains is exclusively DH-KR [22]. The only exception can be seen in the PKS genes for enediynes in which the chain elongation is iteratively catalyzed as similar to type II PKS [23]. The unusual order of KR-DH may render an undescribed function to the DH domain of RakEF. After formation of APDA moiety, RakG is likely responsible for the condensation of glycine and the following *N*-methylation, and RakH for asparagine condensation. Hydroxylation of asparagine would be catalyzed by asparagine hydroxylase encoded by *rakO* in the downstream of the cluster, to yield rakicidin D. On the basis of the above-mentioned bioinfomatic evidences, we here propose the biosynthetic pathway of rakicidin D as shown Fig. 3.

**Table 6:** ORFs in the rakicidin-biosynthetic gene cluster of *Streptomyces* sp. MWW064

ORF (locus tag)	Size (aa)	Deduced function	Protein homolog [origin]	Identity/similarity (%)	Accession number
SSP35_09_01970	243	unknown	hypothetical protein [*Streptomyces natalensis*]	63/73	WP_030067339
SSP35_09_01960	331	transcriptional regulator	hypothetical protein DT87_01625 [*Streptomyces* sp. NTK 937]	59/69	KDQ65969
SSP35_09_01950	358	3-oxoacyl-ACP synthase	3-oxoacyl-ACP synthase [*Streptomyces* sp. NRRL S-920]	77/87	WP_030791445
SSP35_09_01940	79	ACP	phosphopantetheine-binding protein [*Streptomyces bingchenggensis* BCW-1]	68/79	ADI05068
SSP35_09_01930	406	ketosynthase	3-oxoacyl-ACP synthase [*Streptomyces* sp. NRRL S-15]	81/89	WP_031089521
SSP35_09_01920	146	unknown	methylmalonyl-CoA epimerase [*Salinispora pacifica*]	80/87	WP_018222873
SSP35_09_01910 (RakAB)	2,902	PKS	hypothetical protein [*Streptomyces vitaminophilus*]	63/73	WP_018385948
SSP35_09_01900 (RakC)	1,624	PKS	non-ribosomal peptide synthetase [*Micromonospora* sp. M42]	60/70	EWM63000
SSP35_09_01890 (RakD)	1,126	NRPS	hypothetical protein [*Streptomyces vitaminophilus*]	68/78	WP_018385946
SSP35_09_01880 (RakEF)	1,950	PKS	hypothetical protein [*Streptomyces vitaminophilus*]	64/73	WP_018385945
SSP35_09_01870 (RakG)	1,556	NRPS	hypothetical protein, partial [*Micromonospora purpureochromogenes*]	64/74	WP_030498976
SSP35_09_01860 (RakH)	1,565	NRPS	amino acid adenylation domain protein [*Nostoc punctiforme* PCC 73102]	38/55	ACC80782
SSP35_09_01850	563	ABC transporter	hypothetical protein [*Micromonospora purpureochromogenes*]	62/73	WP_030498978
SSP35_09_01840 (RakL)	251	type-II thioesterase	hypothetical protein [*Streptomyces vitaminophilus*]	64/73	WP_018385940
SSP35_09_01830 (RakN)	1,013	NRPS	non-ribosomal peptide synthetase [*Micromonospora* sp. M42]	55/63	EWM63010
SSP35_09_01820 (RakO)	331	asparagine oxygenase	clavaminate synthase [*Streptomyces* sp. LaPpAH-202]	64/75	WP_026235187
SSP35_09_01810	809	unknown	penicillin amidase [*Amycolatopsis nigrescens*]	63/74	WP_026359955
SSP35_09_01800	205	transcriptional regulator	putative LuxR family transcriptional regulator [*Streptomyces glaucescens*]	71/81	AIR96926

SSP35_09_01910, SSP35_09_01900, SSP35_09_01890, SSP35_09_01880, SSP35_09_01870, SSP35_09_01860, SSP35_09_01840, SSP35_09_01830, and SSP35_09_01820 are corresponding to RakA plus RakB (RakAB), RakC, RakD, RakE plus RakF (RakEF), RakG, RakH, RakL, RakN, and RakO, previously reported in the reference [8], respectively, and SSP35_09_01940 may possibly be corresponding to RakI

## Identification of biosynthetic precursors of the APDA moiety

**Fig. 4:**
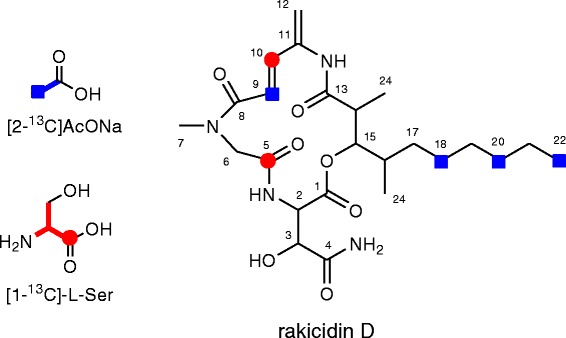
Incorporation of ^13^C-labeled precursors into rakicidin D

To verify the predicted biosynthetic origin of the APDA unit, feeding experiments using ^13^C-labeled precursors were carried out. Inoculation, cultivation, extraction, and purification were performed in the same manner as previously reported [1]. Addition of sodium [2-^13^C]acetate or [1-^13^C]-L-serine (20 mg/100 ml medium/flask, 10 flasks for [2-^13^C]acetate, 3 flasks for [1-^13^C]-L-serine) was initiated at 48 h after inoculation and periodically carried out every 24 h for four times. After further incubation for 24 h, the whole culture broths were extracted with 1-butanol and several steps of purification yielded 2.5 mg and 1.7 mg of ^13^C-labeled rakicidin D, respectively. The ^13^C NMR spectrum of these labeled rakicidin D is shown in Table 7. Feeding of sodium [2-^13^C]acetate gave enrichments at C9 of the APDA unit and three carbons C18, C20, and C22 in the aliphatic chain of the fatty acid moiety. [1-^13^C]-L-serine feeding enriched C10 of the APDA unit and the carbonyl carbon of Gly (C5). These results unambiguously indicated that the APDA unit is derived from an acetate and a serine (Fig. 4). Labeling of C5 by serine-feeding can be explained by the interconversion between glycine and serine by transformylase in primary metabolism for amino acid supply.

**Table 7:** Incorporation of ^13^C-labeled precursors into rakicidin D

Position	δ_C_	Relative enrichments^a^	
		[2-^13^C]acetate	[1-^13^C]-L-serine
1	169.2	0.89	1.58
2	54.9	1.14	1.19
3	72.5	1.57	1.07
4	172.7	1.31	1.95
5	167.6	0.68	**6.61**
6	52.5	0.77	1.15
7	36.5	1.00	1.00
8	166.0	0.77	1.43
9	118.8	3.46	1.54
10	138.4	1.03	**13.97**
11	137.9	0.95	0.67
12	117.1	1.02	1.33
13	172.5	1.61	1.26
14	41.7	1.86	1.36
15	78.1	1.25	1.21
16	33.9	1.91	1.39
17	32.8	1.30	1.55
18	27.0	**2.64**	1.26
19	28.9	1.15	1.58
20	31.3	**3.37**	1.43
21	22.1	1.05	1.28
22	14.0	**3.37**	1.61
23	15.4	1.51	1.07
24	13.3	1.75	1.32

^a^ ^13^C signal intensity of each peak in the labeled **1** divided by that of the corresponding signal in the unlabeled **1**, respectively, normalized to give an enrichment ratio of **1** for the unenriched peak of C7. The numbers in bold type indicate ^13^C-enriched atoms from ^13^C-labeled precursors

## Conclusions

The 7.9 Mb draft genome of *Streptomyces* sp. MWW064, a producer of rakicidin D isolated from marine segment, has been deposited at GenBank/ENA/DDBJ under the accession number BBUY00000000. We successfully identified the PKS/NRPS hybrid gene cluster for rakicidin synthesis and proposed the plausible biosynthetic pathway. Labeled precursor incorporation experiments showed the APDA moiety is synthesized from serine and malonate. These finding will open up possibilities of genetic engineering to synthesize more potential rakicidin-based antitumor compounds and discovering new bioactive compounds possessing APDA units.

## Abbreviations

A: Adenylation
ABC: ATP-binding cassette
ACP: Acyl carrier protein
APDA: 4-amino-2,4-pentadienoate
AT: Acyltransferase
C: Condensation
CoA: Coenzyme A
CoL: CoA ligase
DDBJ: DNA Data Bank of Japan
DH: Dehydratase
ER: Enoylreductase
ISP: International *Streptomyces* project
KR: Ketoreductase
KS: Ketosynthase
NBRC: Biological Resource Center, National Institute of Technology and Evaluation
NMR: Nuclear magnetic resonance
NRPS: Nonribosomal peptide synthetase
PKS: Polyketide synthase
T: Thiolation
